# Correction: SynAsk: unleashing the power of large language models in organic synthesis

**DOI:** 10.1039/d6sc90209j

**Published:** 2026-10-28

**Authors:** Chonghuan Zhang, Qianghua Lin, Biwei Zhu, Haopeng Yang, Xiao Lian, Hao Deng, Jiajun Zheng, Kuangbiao Liao

**Affiliations:** a Guangzhou National Laboratory Guangzhou Guangdong 510005 PR China liao_kuangbiao@gzlab.ac.cn; b AIChemEco Inc. Guangzhou Guangdong 510005 PR China

## Abstract

Correction for ‘SynAsk: unleashing the power of large language models in organic synthesis’ by Chonghuan Zhang *et al.*, *Chem. Sci.*, 2025, **16**, 43–56, https://doi.org/10.1039/d4sc04757e.

Following discussions with the editorial office, the authors have expanded their Conflicts of interest and Acknowledgements sections to ensure that they are as detailed as possible. The full statements are available below.

**Conflicts of interest**

We have a patent application in China with the application number 202410714040.6 titled “A human–computer interaction method and electronic device based on a large language model”.

K. L. is the founder and Chief Scientist of AIChemEco Inc. and holds equity in the company. B. Z., H. Y., X. L., H. D. and J. Z. are employees of AIChemEco Inc. AIChemEco Inc. developed and operates the SynAsk platform and owns or controls the associated software, data resources and related intellectual property. The company also provided personnel and in-kind support for this study, including software and computational resources, data-related work, platform development and deployment infrastructure, and associated technical support. The other authors declare no competing interests.

**Acknowledgements**

We are grateful for financial support from Guangzhou National Laboratory and the National Natural Science Foundation of China (22071249, 22393892).

We also acknowledge in-kind support from AIChemEco Inc., including personnel time, software and computational resources, data-related work, platform development and deployment infrastructure, and associated technical support.

The Royal Society of Chemistry apologises for these errors and any consequent inconvenience to authors and readers.

